# Editorial: Anesthetic Risk and Complications in Veterinary Medicine

**DOI:** 10.3389/fvets.2020.00397

**Published:** 2020-07-15

**Authors:** Karine Portier, Keila K. Ida

**Affiliations:** ^1^Université de Lyon, VetAgro Sup, GREAT, Marcy l'Etoile, France; ^2^Université de Lyon, CarMeN Laboratory, INSERM, INRA, INSA Lyon, Université Claude Bernard Lyon 1, Bron, France; ^3^Department of Small Animal Clinical Sciences, College of Veterinary Medicine and Biomedical Sciences, Texas A&M University, College Station, TX, United States

**Keywords:** death, morbidity and mortality, anesthesia, animals, safety

Veterinary practitioners have obligations to inform owners of the potential risks their animal might encounter during a surgery. A third of veterinarians believe that the majority of their clients are particularly concerned about their animal being anesthetized. The lack of a clear definition of anesthesia-related mortality and morbidity makes it difficult to specify the real anesthetic risk to the animals' owners. The timing a complication occurs, intra- or postoperatively, can also impose uncertainty in defining whether incidents are associated with the anesthetic procedure.

Large veterinary multicenter studies defined anesthesia-related death as those occurring within 48 h (small animals) or 7 days (horses) of termination of the procedure, where anesthesia could not be excluded as being one of the contributory factors. Based on this definition, the authors identified an overall 0.17% anesthetic-related risk of death in dogs, 0.24% in cats, and 1.9% in horses. Such high rates compared with human patients warned clinicians and researchers on the need of improvements. Since then, several efforts have been made to increase the safety of animals undergoing anesthesia. This Research Topic was part of these efforts by creating an opportunity for the contribution of 35 researchers through 12 publications on the subject. They share, among others, the challenges found on the attempts to prevent the occurrence of deaths and complications. They also describe clinical complications and the successful management that was applied.

Since 2002, when it was announced that horses have a high mortality rate associated with anesthesia, new equipment was developed to improve safety in this animal species. In 2008, Tafonius, the large animal anesthesia machine, was released with integrated monitoring and ventilator systems. The emerging technology allowed, among other features, to control the fresh gas flow into the breathing system either by a manually- or computer-driven flowmeter. The convenience of having a machine adjusting the flow of different gases to pre-determined concentrations is an attractive feature and its accuracy was, therefore, tested by Raillard et al.. In this original article, the authors describe that the prediction of the isoflurane fraction course in the breathing system was challenging when using the computer-driven flowmeter. This was especially true at low inspired fractions of oxygen. The discrepancies between flows set on the controlled-driven flowmeter and actual lower delivered flows should be taken into consideration. Insufficient concentrations of inhalant anesthetics might lead to serious safety concerns, including both awaking of horses during anesthesia or unwarranted high concentrations of anesthetics that might result in cardiovascular and respiratory complications.

An excessive delivery of inhalant anesthetics can significantly decrease the systemic vascular resistance and cause relative hypovolemia. This is of particular concern in equine patients since 20–50% of all anesthesia-related deaths in this animal species are associated with cardiovascular complications. The exact mechanisms are explained by Noel-Morgan and Muir who also provide further considerations on the monitoring and treatment of anesthesia-associated relative hypovolemia. Such perspective is especially important in life-threatening situations, which become evident in three of the clinical cases reported in the present Research Topic. Tong et al. describe the management of recurrent hyperkalemia during general anesthesia in a dog. Marolf et al. describe the development of an advanced atrio-ventricular block unresponsive to antimuscarinic drugs in an anesthetized foal. Conde-Ruiz and Junot share the case of a horse suffering a cardiac arrest at the arrival of the recovery room. In all three cases, the authors discuss the potential causes, the preventive measures and the successful treatments applied. They demonstrate how the close monitoring proved to be decisive for early recognition and prompt management of the cardiovascular complications, which were crucial for the good outcome.

A less close monitoring may be responsible for the increased risk of anesthetic-related morbidity and mortality associated with the recovery period. In dogs, cats, and rabbits, nearly 50% of the postoperative deaths occur within 3 h of the end of anesthesia. In horses, Laurenza et al. found that 92% of complications occur during recovery and most of them are associated with neuromuscular and respiratory causes. The pathogenesis of certain conditions is still unclear, which makes it difficult not only to provide adequate care but also to prevent complications in future cases. This was the key point of discussion in the case reported by Mirra et al.. The authors describe an unusual presentation of a potential post-anesthetic neuropathy in the non-dependent limb of a horse. In another case report, Dupont et al. discuss the role of hypoxemia as the potential cause of the delayed recovery from anesthesia in a draft horse. In both occasions, despite of the unknown mechanisms for the clinical alterations, the horses were successfully managed. In some situations, it is possible to anticipate the development of potential postoperative complications and apply preventive measures. This was addressed in the case report by Ida et al. who shared the ventilatory management that prevented respiratory complications during recovery from anesthesia of two ponies with tracheal collapse.

Preventive measures may play an important role to avoid further complications. The reduction of mortality and morbidity risks has been suggested with the use of safety checklists. The implementation of checklists may have some challenges that are revealed in the original research from Menoud et al.. The authors describe numerous methods used to create and implement a safety checklist in a veterinary university teaching hospital. Morbidity and mortality conferences also seem to have an impact on patient care. In the mini review by Pang et al. the authors illustrate the measurable improvements in patient care generated by these conferences, which may also represent a powerful educational tool.

Another preventive tool is the ASA PS classification, which ability to identify animals at a greater risk of anesthesia-related death was put in question in the systemic review by Portier and Ida. The authors assessed a total of 258,298 dogs, cats, rabbits, and pigs. The results show evidences to justify the use of the ASA PS as a prognostic tool to identify the odds of death related to anesthesia in these animal species. In fact, Laurenza et al. identified that a high ASA PS score represents, among other factors, a major risk for mortality and complications in horses. In this original research article, the authors describe a 1.4% overall mortality associated with anesthesia on horses in a French university teaching hospital. It corresponds to a reduction from the 1.9% anesthesia-related equine deaths announced in 2002. Now, 18 years later, the effects of several efforts to improve case management and decrease the morbidity and mortality seem to have had a positive impact.

We believe that this Research Topic adds to the clinical practice of veterinary doctors, contributing to reduce the anesthetic risk and complications in animals. We also expect that this Research Topic motivates readers to share their experience with anesthetic complications and to produce further studies in this field. This, combined with the implementation of expert recommendations, could contribute to the continuing improvement of the quality of anesthesia and, therefore, to decrease the complications and the mortality rate of veterinary patients undergoing anesthesia.

## Author Contributions

KP and KI co-edited the Research Topic and wrote this editorial. All authors contributed to the article and approved the submitted version.

## Conflict of Interest

The authors declare that the research was conducted in the absence of any commercial or financial relationships that could be construed as a potential conflict of interest.

